# A cross-species genetic analysis identifies candidate genes for mouse anxiety and human bipolar disorder

**DOI:** 10.3389/fnbeh.2015.00171

**Published:** 2015-07-01

**Authors:** David G. Ashbrook, Robert W. Williams, Lu Lu, Reinmar Hager

**Affiliations:** ^1^Computational and Evolutionary Biology, Faculty of Life Sciences, University of ManchesterManchester, UK; ^2^Department of Genetics, Genomics and Informatics, University of Tennessee Health Science Center, University of TennesseeMemphis, TN, USA; ^3^Jiangsu Key Laboratory of Neuroregeneration, Nantong UniversityNantong, China

**Keywords:** bipolar disorder, *TNR*, *CMYA5*, *RXRG*, *MCTP1*, anxiety, cross-species

## Abstract

Bipolar disorder (BD) is a significant neuropsychiatric disorder with a lifetime prevalence of ~1%. To identify genetic variants underlying BD genome-wide association studies (GWAS) have been carried out. While many variants of small effect associated with BD have been identified few have yet been confirmed, partly because of the low power of GWAS due to multiple comparisons being made. Complementary mapping studies using murine models have identified genetic variants for behavioral traits linked to BD, often with high power, but these identified regions often contain too many genes for clear identification of candidate genes. In the current study we have aligned human BD GWAS results and mouse linkage studies to help define and evaluate candidate genes linked to BD, seeking to use the power of the mouse mapping with the precision of GWAS. We use quantitative trait mapping for open field test and elevated zero maze data in the largest mammalian model system, the BXD recombinant inbred mouse population, to identify genomic regions associated with these BD-like phenotypes. We then investigate these regions in whole genome data from the Psychiatric Genomics Consortium's bipolar disorder GWAS to identify candidate genes associated with BD. Finally we establish the biological relevance and pathways of these genes in a comprehensive systems genetics analysis. We identify four genes associated with both mouse anxiety and human BD. While *TNR* is a novel candidate for BD, we can confirm previously suggested associations with *CMYA5*, *MCTP1*, and *RXRG*. A cross-species, systems genetics analysis shows that *MCTP1*, *RXRG*, and *TNR* coexpress with genes linked to psychiatric disorders and identify the striatum as a potential site of action. *CMYA5*, *MCTP1*, *RXRG*, and *TNR* are associated with mouse anxiety and human BD. We hypothesize that *MCTP1*, *RXRG*, and *TNR* influence intercellular signaling in the striatum.

## Introduction

Bipolar disorder (BD) is a neuropsychiatric disorder characterized by recurrent periods of mania and depression. Bipolar I disorder has a lifetime prevalence (i.e., at least one case of both depression and mania) of ~1%, although the bipolar spectrum (at least one episode of sub-threshold mania or hypomania) has a lifetime prevalence of up to 6.4% (Judd and Akiskal, 2003; Merikangas et al., 2007). Twin studies estimate heritability of ~60–80%, indicating a substantial genetic component (McGuffin et al., 2003; Edvardsen et al., 2008; Lichtenstein et al., 2009; Wray and Gottesman, 2012).

To identify genetic variants underlying the disorder many genome-wide association studies (GWAS) have been conducted showing that BD is highly polygenic, with many single nucleotide polymorphisms (SNPs) each of small effect (Ferreira et al., 2008; Purcell et al., 2009; Scott et al., 2009; Psychiatric GWAS Consortium Bipolar Disorder Working Group, 2011). Due to this polygenic nature large sample sizes are required to detect SNPs with genome-wide significance. Indeed, despite large cohorts of patients used, GWAS have found only 10 SNPs which are strongly and consistently associated with the disorder (Szczepankiewicz, 2013; Mühleisen et al., 2014), although many genes have been identified with lower confidence or using additional analyses. For example, a recent pathway analysis study has linked 226 genes to BD (Nurnberger et al., 2014), however, approximately 5% of these would be expected to be false positives and the method is biased against genes of small size (Nurnberger et al., 2014). In order to understand the etiology and biology of bipolar disorder it is critical to know the underlying causal variants. This allows us to link the disorder to specific proteins and pathways, potentially leading to novel treatments and a better ability to predict genetic predisposition.

GWAS in humans typically have modest statistical power due to high multiple testing corrections. However, loci are defined with high precision, often to the individual SNP level. By contrast, mouse linkage studies often have high statistical power to detect genetic effects but lower resolution, producing loci that include tens or hundreds of genes (Mackay et al., 2009; Ackert-Bicknell et al., 2010; Hager et al., 2012; Wu et al., 2014). Combining data from mice and humans overcomes some of these problems, gaining power from mouse crosses and precision from human GWAS. This method also ensures translational relevance, as the same gene controlling similar phenotypes is found in a related species (Ashbrook et al., 2014). Moreover, the approach illustrates that the mouse homolog is relevant to the human phenotype, allowing research to be carried out on the gene to phenotype pathway which would not be possible in humans (Kas et al., 2007).

**Figure 1:**
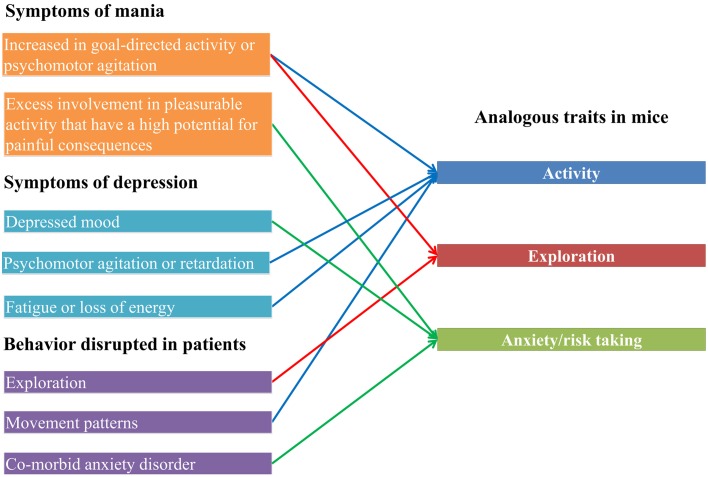
**Bipolar disorder traits and analogous mouse phenotypes**. On the left, symptoms of the manic and depressive phases of bipolar disorder are shown, as well as behaviors which have been demonstrated to be disrupted in bipolar disorder patients. On the right are mouse phenotypes which are measured by the zero maze and open field test, showing how the human traits link to the mouse traits.

Mammalian model systems have been extensively used to investigate the genetic basis of disease traits through the experimental study of analogous behavioral or developmental traits (e.g., Hayes et al., 2014). We chose to investigate BD, as several of the symptoms are related to behaviors which can be measured in mice. For example, in manic patients typical behaviors include motor hyperactivity, increased risk taking and impulsive behavior (Goodwin and Jamison, 1990). Indeed, anxiety and activity measures in mice have previously been used to evaluate animal models of BD (Gould et al., 2001; Kirshenbaum et al., 2011). Furthermore, up to 93% of bipolar I disorder patients have a comorbid anxiety disorder at some stage of their life, and comorbidity between BD and anxiety results in significantly worse patient outcomes (Freeman et al., 2002; MacKinnon, 2002; Boylan et al., 2004; Simon et al., 2004; Merikangas et al., 2007; Goldstein and Levitt, 2008; Goodwin and Sachs, 2010; Vázquez et al., 2014). Further, measures of global anxiety correlate well with time spent in depressive episodes in bipolar patients (Coryell et al., 2009). This suggests a shared underlying etiology of anxiety and BD. Therefore, there is a clear relationship between symptoms of bipolar disorder (Goodwin and Sachs, 2010), behaviors disrupted in bipolar patients (Young et al., 2007; Perry et al., 2009; Minassian et al., 2011) and measureable behaviors in mice (Figure 1).

Most organisms will balance risk against potential reward; whether to put greater emphasis on minimizing exposure to danger (being more anxious), or greater emphasis on reward (being more risk taking) (Marks and Nesse, 1994; Bateson et al., 2011). This trade-off is reflected in a number of behavioral patterns, and individuals in a population differ in the degree to which anxiety-like behavior is displayed (Erhardt and Spoormaker, 2013). Since normal and clinical anxiety exist upon a continuum (Bateson et al., 2011) and aspects of pathological anxiety and BD overlap in terms of etiology and phenotype, it is reasonable to assume that some aspects of the underlying anxiety system should also overlap with BD, although true anxiety is more easily segregated from bipolar disorder in humans.

Previous studies demonstrated the utility of using a cross-species approach to identify genes underlying specific traits (De Mooij-van Malsen et al., 2009; Koutnikova et al., 2009; Leduc et al., 2011; Poot et al., 2011; Schofield et al., 2012; Ashbrook et al., 2014). This approach has the advantage that it allows the investigation of phenotypes without requiring experimental perturbations. We utilize data obtained from populations that segregated for large numbers of common sequence variants and associated differences in phenotype (Ashbrook et al., 2014). Here, we use data for two common measures of anxiety and activity in mouse, the elevated zero maze and the open field test, to identify QTL in the largest mammalian model system, the recombinant inbred mouse panel BXD (Wu et al., 2014). This identifies areas of the genome containing genetic variants which influence BD-like phenotypes in mice, and therefore may be influencing aspects of BD in humans. These genomic regions were then investigated in a large human GWAS of BD, the SNP summary of which has been made available online (Psychiatric GWAS Consortium Bipolar Disorder Working Group, 2011). We identify four genes (*TNR, RXRG, MCTP1*, and *CMYA5)* associated with anxiety in mice and risk of BD in humans. A systems genetics approach suggests that *TNR, RXRG*, and *MCTP1* coexpress with several other genes related to mental disorders in the striatum, providing a potential mechanism of action.

## Methods and materials

### Mouse and human data

The BXD recombinant inbred population consists of experimentally tractable and genetically defined mouse lines capturing a large amount of naturally occurring genetic variation, which underlies variation at the phenotypic level (e.g., Chesler et al., 2005; Mozhui et al., 2011; Hager et al., 2012). Over 140 BXD lines incorporate ~5 million segregating SNPs, 500,000 insertions and deletions, and 55,000 copy-number variants (Mozhui et al., 2011). These lines are used for complex systems genetics analyses integrating massive phenotype and gene expression data sets obtained in different studies (Andreux et al., 2012; Hayes et al., 2014). For our analysis we used data for two murine phenotypes, the elevated zero maze and open field test (Philip et al., 2010) available in GeneNetwork (genenetwork.org; February 2015) that measure a combination of anxiety, exploration and activity (Henry et al., 2010), all of which are altered in BD (Goodwin and Jamison, 1990; Young et al., 2007). We selected traits with at least one significant QTL (*p* ≤ 0.05) at the genome-wide level. Significance was calculated within GeneNetwork, using 5000 permutations of trait values and genotypes. A *p*-value of 0.05 is defined as an LRS score greater than 95% of the permuted datasets (Wang et al., 2003).

Human GWAS data for BD were obtained from the Psychiatric Genomics Consortium (PGC; https://pgc.unc.edu), containing 11,974 BD cases and 51,792 controls (Psychiatric GWAS Consortium Bipolar Disorder Working Group, 2011). The majority of cases were of BD type 1 although also included BD type 2, schizoaffective disorder bipolar type and individuals with other bipolar diagnoses (Psychiatric GWAS Consortium Bipolar Disorder Working Group, 2011). Knowledge-Based Mining System for Genome-wide Genetic Studies (KGG; http://statgenpro.psychiatry.hku.hk/limx/kgg; version 3.5) was used to convert these SNP *p*-values to gene *p*-values, using both the GATES and the hybrid set-based test (HYST) methods (Li et al., 2010, 2011, 2012). The GATES test is a Simes test extension that is valid for correlated SNPs, and, of the two methods, is more powerful for genes with one or few independent causal variants (Li et al., 2011). HYST combines the GATES test and the scaled chi-square test (Moskvina et al., 2011) to examine the overall association significance in a set of SNPs. This test is more powerful for genes with a number of independent causal variants. Both tests are advantageous as they require only summary GWAS and ancestral population linkage disequilibrium data, rather than raw data. Gene locations were from the Hg18 genome build. We used the complete set of data from the Psychiatric Genomics Consortium GWAS for BD (http://www.med.unc.edu/pgc/downloads; pgc.bip.full.2012-04.txt), and linkage disequilibrium data from the CEPH (Utah residents with ancestry from northern and western Europe; CEU) Hapmap population dataset (http://hapmap.ncbi.nlm.nih.gov/downloads/ld_data/?N=D). For each gene in the Hg18 genome build a GATES and HYST *p*-value was calculated for associated with bipolar disorder.

For the joint mouse—human analysis, the human homologs of genes within the mouse QTL were identified using Homologene (http://www.ncbi.nlm.nih.gov/homologene). To assess if any particular gene is associated with both anxiety in mouse and BD, we examined both the GATES derived and HYST derived *p*-values of human homologs of genes within the significant BXD QTL. The human GWAS significance values were Bonferroni corrected for multiple comparisons using the total number of homologous genes compared and the number of tests used (two: HYST and GATES) (De Mooij-van Malsen et al., 2009; Ashbrook et al., 2014).

### Evolutionary conservation

A highly conserved nucleotide sequence between mouse and human would support that the protein plays the same role in both species. For each gene the protein it codes for was identified using the Entrez gene database (http://www.ncbi.nlm.nih.gov/gene) and the NCBI reference sequence for the protein identified. For each pair of homologous proteins a Protein BLAST was carried out with default settings (http://blast.ncbi.nlm.nih.gov/Blast.cgi?PROGRAM=blastp&PAGE_TYPE=BlastSearch&LINK_LOC=blasthome). The reference sequences used were: RXRG human NP_001243499.1, mouse NP_001153203.1; TNR human NP_003276.3, mouse NP_071707.2; MCTP1 human NP_001002796.1, mouse NP_084450.2; CMYA5 human NP_705838.3, mouse NP_076310.2.

### Identification of areas of expression

To identify the regions in the mouse brain in which our candidate genes are expressed we used images provided by the Allen Brain Atlas showing patterns of gene expression (http://mouse.brain-map.org; June 2015) throughout the adult mouse brain for >20,000 genes, using *in situ* hybridization (Lein et al., 2007). For the human data, the BrainSpan Atlas of the Developing Human Brain (Miller et al., 2014) contains RNA-*seq* gene expression data at different life stages. There are a total of 578 samples, covering an age range from prenatal to 40 years and taken from 26 different brain regions. To identify locations in the human brain where our candidate genes are expressed we used gene expression heatmaps that show levels of gene expression in different brain regions (http://www.brainspan.org; June 2015).

### “Guilt-by-association”

To establish if specific genes are coexpressed in humans we used GeneFriends (Van Dam et al., 2012; http://genefriends.org/microArray/; January 2014) which contains microarray data (4164 microarray datasets containing 26,113 experimental conditions and 19,080 genes) from the Gene Expression Omnibus (Van Dam et al., 2012; Barrett et al., 2013). This enabled us to find genes that are commonly coexpressed (i.e., genes with a coexpression value ≥ 0.50 and a *p* ≤ 0.05) with a submitted gene list. A coexpression value of 0.50 indicates that a particular gene is increased in expression at least two-fold in 50% of the cases that a target gene is increased in expression at least two-fold.

However, GeneFriends is not tissue or treatment specific, and therefore can only show the genes that are coexpressed together, not when or where. This common coexpression suggests that the genes are under the same regulation. The list of commonly coexpressed genes was analyzed using WebGestalt, producing annotations for these genes. This “guilt-by-association” approach enables us to identify the biological networks of our candidates (Van Dam et al., 2012). WebGestalt (http://bioinfo.vanderbilt.edu/webgestalt/; May 2014) is a web-based enrichment analysis tool that incorporates information from online sources including Gene Ontology (GO), KEGG pathways, Wikipathways, Pathways Commons, and disease association analysis (Zhang et al., 2005; Wang et al., 2013). The lists of genes generated by our “guilt-by-association” analysis were submitted to WebGestalt to identify pathways or diseases that our candidate genes may be involved in. Significance of enrichment was determined by the Benjamini and Hochberg method of multiple test adjustment (Benjamini and Hochberg, 1995) as implemented in WebGestalt, and the whole human genome was used as the background set of genes.

Shared function of genes can be established using coexpression analysis (Allocco et al., 2004). We analyzed coexpression in adult mouse brain by producing Pearson product-moment correlation matrices of striatal and hippocampal gene expression (Chesler et al., 2003) as implemented in GeneNetwork. The striatum was chosen as it is an area where several of our genes are expressed, whereas the hippocampus was chosen to identify if any coexpression seen in the striatum was seen in other brain regions. Expression data was obtained from GeneNetwork HQF Striatum Exon (Feb09) RMA data (GN163) and UMUTAffy Hippocampus Exon (Feb09) RMA (GN206). Since both used the same microarray we could directly compare the data. Probes for exons were used unless no exon probes were available, in which case introns were investigated for consistent coexpression within a given gene. Coexpression between our candidate genes and genes previously associated with BD (as identified in Szczepankiewicz, 2013; Mühleisen et al., 2014), and other mental disorders was calculated in GeneNetwork using Pearson correlations. Multiple comparisons were corrected for by dividing the *p*-value obtained in the correlation analysis by the number of probes for our genes of interest (*Cmya5, Mctp1, Rxrg, Tnr*). Probes were said to coexpress if they have an *r* ≥ 0.5 or ≤ −0.5 and an adjusted *p* ≤ 0.05. This allowed us to establish if our candidate genes coexpress with genes known to be associated with neuropsychiatric disorders, and whether this is specific to the striatum.

### Principal component analysis

Principal component analysis (PCA) was used to jointly analyze multiple phenotypes including gene expression. PCA reduces the dimensionality of data and captures the shared variability between traits. If the first principal component (PC1) explains a high proportion of the variability it can be used as a synthetic trait, capturing the main common source of variation within the traits (Mozhui et al., 2011). PCA was carried out in GeneNetwork to find the PC1 of striatal expression of our candidate gene *MCTP1* and the PC1 of the open field test phenotypes.

### Biological networks

In order to establish the biological networks of our candidate genes we utilized the massive phenotype and expression data sets available for the BXD panel (Gini and Hager, 2012; Ashbrook and Hager, 2013). Generally, microarray analysis uses a number of probes, targeted at different parts of a gene. Exon level microarrays, such as those used above in the coexpression analysis, can be used to calculate expression of exons, introns or untranscribed regions (UTRs). Consistent correlation between different probes for a gene and a phenotype would suggest that the gene is associated with the phenotype.

First, using the same exon level data as used for the coexpression analysis, probes for each of our four candidate gene were examined for high Pearson correlation (*r* ≥ 0.5, *p* ≤ 0.05). For each gene, this produced a group of highly correlated probes. Each of these highly correlated probes was individually correlated against BXD phenotypes, which yielded a list of phenotypes that correlated with that probe (*r* ≥ 0.5 or ≤ −0.5 and *p* ≤ 0.05). Next, the phenotypes which correlated with all the probes for a particular candidate gene were identified, to produce a smaller list of phenotypes which consistently correlated with all the probes for that gene.

## Results

**Figure 2:**
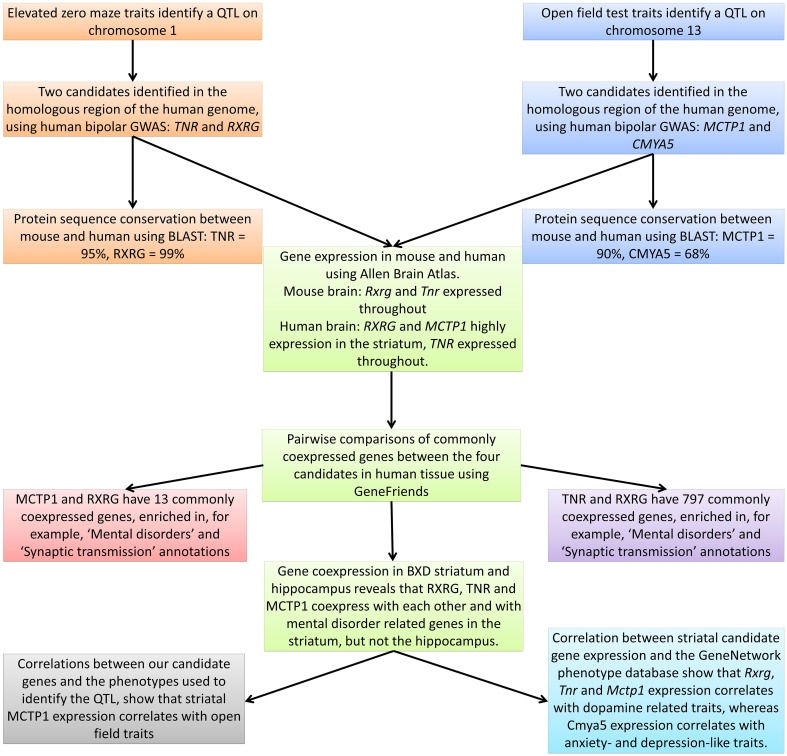
**Graphical representation of the research method used, providing a summary of the main findings**.

In Figure 2 we give a graphical summary of our results. We begin by identifying QTL for the open field test and elevated zero maze in the BXD recombinant inbred lines. We then investigate the homologous genomic regions in human bipolar disorder GWAS data and thus identify four candidate genes (*TNR*, *RXRG, MCTP1*, and *CMYA5*). The protein products of three of these genes (*TNR, RXRG*, and *MCTP1*) are highly conserved (≥90% identical), and are expressed in the adult striatum of both mouse and human. In non-tissue specific human gene expression data these three genes coexpress with genes that are associated with mental disorders. In the BXD mouse striatum the three genes coexpress together, as well as with known mental disorder related genes. Finally, when we correlate striatal expression of all four of our candidate genes against the large BXD phenotype dataset, we find that *Tnr, Rxrg*, and *Mctp1* expression correlates with dopamine related traits, whereas *Cmya5* expression correlates with anxiety- and depression-like traits.

### QTL for bipolar related phenotypes in mice

Bipolar disorder is characterized by disrupted anxiety, activity and exploration (Goodwin and Jamison, 1990; Goodwin and Sachs, 2010; Figure 1) and we thus investigated genetic variation in those traits, focusing on the elevated zero maze and the open field test. Both of these are used to measure anxiety, activity and exploration in mice (Shepherd et al., 1994; Chauhan et al., 2005; Kalueff et al., 2006; Ariyannur et al., 2013). We limited the datasets to only those with no or saline only treatment, and with at least one genome-wide significant QTL (*p* ≤ 0.05).

**Figure 3:**
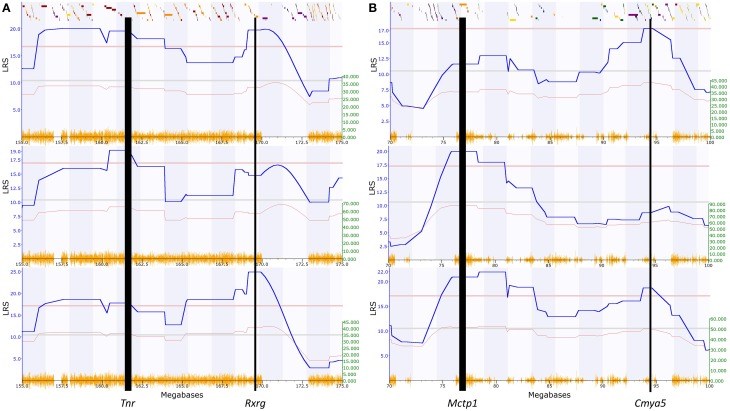
**Example QTL maps showing two significant QTL within each of the regions we identified. (A)** Chromosome 1, between 155 and 175 Mbp, elevated zero maze traits (GeneNetwork ID 12419, 12361 and 12409; full details in Table 1). **(B)** Chromosome 13, between 70 and 100 Mbp, open field test traits 11606, 11759, 11607 (Full details in **Table 2**). This clearly shows that some traits have two peaks, with a non-significant region between, while other traits have significant peaks only at one end or the other of the identified regions. The higher red line indicates the level of genome-wide significance, i.e., a genome-wide corrected *p* ≤ 0.05, with the blue line showing the significance of the trait at each position. The lower red line indicates the negative additive coefficient, i.e., that C57BL/6J alleles increase trait values, with the scale in green on the left. The colored blocks at the top of the figure show the positions of genes. The orange track at the bottom of each map is the SNP Seismograph track, showing the positions of SNPs within the BXD lines. The location of our four candidate genes are shown, with the width of the line representing the size of the gene.

For the seven available elevated zero maze traits, QTL were consistently found on distal chromosome 1 (156.2–175.3 Mbp), containing 185 genes with human homologs (Table 1, Supplementary Figure 1). Therefore, the significance threshold for human genes is *p* = 1.4e-4 (185 genes, each compared against both the GATES and HYST significance values; 0.05/185^*^2). However, the size of the above region differed between the datasets, and appears to separate into two smaller QTL, based on phenotypes showing either two peaks, with a non-significant region between them, or a significant peak at only one of the two QTL (Figure 3A; Supplementary Table 1; Supplementary Figure 1).

**Table 1:** **Activity traits in the elevated zero maze which have a significant QTL**.

**ID**	**Phenotype**	**Number of lines phenotyped**	**Number of genes**	**Position of genes (mm9)**
12359	Central nervous system, behavior: Anxiety assay, baseline untreated control (BASE group), activity in closed quadrants using an elevated zero maze in 60–120-day-old males and females during first 5 min [n beam breaks]	74	3	Chr1:160.6–161.5
12361	Central nervous system, behavior: Anxiety assay, baseline untreated control (BASE group), activity in closed quadrants using an elevated zero maze in 60–120-day-old males and females during 10 min [n beam breaks]	74	8	Chr1:160.6–162.2
12409	Central nervous system, behavior: Anxiety assay, saline treated [0.18 ml/kg i.p.] (NOS group), activity in closed quadrants using an elevated zero maze in 60–120-day-old males only during first 5 min [n beam breaks]	73	30	Chr1:156.2–161.5
			44	Chr1:165.3–171.0
12411	Central nervous system, behavior: Anxiety assay, saline treated [0.18 ml/kg i.p.] (NOS group), activity in closed quadrants using an elevated zero maze in 60–120-day-old males only during 10 min [n beam breaks]	73	30	Chr1:156.2–161.5
			7	Chr1:169.1–170.0
12419	Central nervous system, behavior: Anxiety assay, saline treated [0.18 ml/kg i.p.] (NOS group), activity in closed quadrants using an elevated zero maze in 60–120-day-old males and females during first 5 min [n beam breaks]	75	60	Chr1:156.2–164.9
			9	Chr1:169.1–171.5
12420	Central nervous system, behavior: Anxiety assay, saline treated [0.18 ml/kg i.p.] (NOS group), activity in closed quadrants using an elevated zero maze in 60–120-day-old males and females during last 5 min [n beam breaks]	75	31	Chr1:156.2–162.0
12421	Central nervous system, behavior: Anxiety assay, saline treated [0.18 ml/kg i.p.] (NOS group), activity in closed quadrants using an elevated zero maze in 60–120-day-old males and females during 10 min [n beam breaks]	75	61	Chr1:157.8–165.0
			9	Chr1:169.1–171.5

*GeneNetwork ID, phenotype and number of lines phenotyped are from GeneNetwork, number of genes refers to the number of genes within the QTL which have human homologs. Position identifies the chromosome and megabase pair (Mbp) location of the gene on mm9*.

The first QTL is located between 156.2 and 161.5 Mbp containing 30 homologous genes (significance threshold for human genes *p* = 8.3e-4). Only one gene, *TNR*, has a *p*-value lower than this threshold (GATES *p* = 7.48e-4; 161.4 Mbp). The second QTL is between 169.1 and 171.5 Mbp, containing 9 homologous genes, with a *p*-value threshold of ≤ 2.7e-3. Only one gene has a lower *p*-value, *RXRG* (GATES *p* = 9.63e-4; 169.5 Mbp). These two genes are the most significant genes within this chromosome 1 region, and their positions line up well with the LRS peaks in the QTL map (Figure 3A).

Consistent QTL for the 34 open field test phenotypes are found on chromosome 13 (73–97 Mbp), containing 63 homologous genes. The threshold for human genes is thus *p* ≤ 3.96e-4. One gene has a *p*-values lower than this threshold: *CMYA5* (HYST *p* = 1.57e-4; 94.2 Mbp). Interestingly, this larger region again appears to segregate into two smaller QTL (Table 2; Figure 3B; Supplementary Figure 2), the first from 73.3 to 84 Mbp (41 homologous genes, *p*-value threshold = 6.1e-4) and the second from 93.9 to 95.2 Mbp (12 homologous genes, *p*-value threshold = 2.1e-3). This reveals another significant gene, *MCTP1* (HYST *p* = 4.59e-4; 76.5 Mbp). Again, these are the two most significant genes within the region, and their positions match those of the LRS peaks on the QTL maps (Figure 3B).

**Table 2:** **Vertical activity traits in the open field which have a significant QTL**.

**ID**	**Phenotype**	**Number of lines phenotyped**	**Number of genes**	**Position of genes (mm9)**
11350	Central nervous system, behavior: Novel open field behavior, vertical activity (rears) from 15 to 30 min for males [n beam breaks]	63	25	Chr13:74.7–82.0
11351	Central nervous system, behavior: Novel open field behavior, vertical activity (rears) from 30 to 45 min for males [n beam breaks]	63	38	Chr13:74.0–82.0
11352	Central nervous system, behavior: Novel open field behavior, vertical activity (rears) from 45 to 60 min for males [n beam breaks]	63	38	Chr13:74.0–82.0
11355	Central nervous system, behavior: Novel open field behavior, vertical activity (rears) in the center from 0 to 60 min for males [n beam breaks]	63	41	Chr13:73.9–84.0
11606	Central nervous system, behavior: Novel open field behavior, vertical activity (rears) from 0 to 15 for females [n beam breaks]	64	10	Chr1:97.0–107.2
			10	Chr13:93.9–94.9
11607	Central nervous system, behavior: Novel open field behavior, vertical activity (rears) from 15 to 30 min for females [n beam breaks]	64	37	Chr13:75.2–95.2
11608	Central nervous system, behavior: Novel open field behavior, vertical activity (rears) from 30 to 45 min for females [n beam breaks]	64	24	Chr13:75.2–83.7
11609	Central nervous system, behavior: Novel open field behavior, vertical activity (rears) from 45 to 60 min for females [n beam breaks]	64	28	Chr9:73.3–78.0
			26	Chr13:74.7–84.0
11612	Central nervous system, behavior: Novel open field behavior, vertical activity (rears) in the center from 0 to 60 min for females [n beam breaks]	64	38	Chr13:74.7–94.9
11759	Central nervous system, behavior: Novel open field behavior, vertical activity (rears) from 0 to 60 min in the center for females [n beam breaks]	64	17	Chr13:75.8–78.7
11772	Central nervous system, behavior: Novel open field behavior, vertical activity (rears) from15 to 30 min in the center for females [n beam breaks]	64	17	Chr13:75.8–78.7
11773	Central nervous system, behavior: Novel open field behavior, vertical activity (rears) from 30 to 45 min in the center for females [n beam breaks]	64	15	Chr13:75.8–78.4
11784	Central nervous system, behavior: Novel open field behavior, vertical activity (rears) in the periphery from 15 to 30 min for females [n beam breaks]	64	22	Chr13:75.8–82.0
11785	Novel open field behavior, vertical activity (rears) in the periphery from 30 to 45 min for females [n beam breaks]	64	24	Chr13:75.2–83.7
11786	Central nervous system, behavior: Novel open field behavior, vertical activity (rears) in the periphery from 45 to 60 min for females [n beam breaks]	64	26	Chr13:74.7–83.6
11790	Central nervous system, behavior: Novel open field behavior, vertical activity (rears) in the periphery from 0 to 60 min for females [n beam breaks]	64	26	Chr13:74.7–83.6
11793	Central nervous system, behavior: Saline control response (10 ml/kg), open field behavior, vertical activity (rears) 0–60 min after injection for females [n beam breaks/60 min]	64	25	Chr13:74.7–82.0
11806	Central nervous system, behavior: Saline control response (10 mg/kg ip), open field behavior, vertical activity (rears) 0–15 min after injection for females [n beam breaks/15 min]	64	48	Chr13:74.7–96.3
11807	Central nervous system, behavior: Saline control response (10 mg/kg ip), open field behavior, vertical activity (rears) 15–30 min after injection for females [n beam breaks/15 min]	64	17	Chr13:75.2–78.7
11808	Central nervous system, behavior: Saline control response (10 mg/kg ip), open field behavior, vertical activity (rears) 30–45 min after injection for females [n beam breaks/15 min]	64	17	Chr13:75.2–78.7
11809	Central nervous system, behavior: Saline control response (10 mg/kg ip), open field behavior, vertical activity (rears) 45–60 min after injection for females [n beam breaks/15 min]	64	17	Chr13:75.2–78.7
11863	Central nervous system, behavior: Novel open field behavior, vertical activity (rears) from 0 to 15 min for males and females [n beam breaks]	64	8	Chr1:97.0–107.2
			9	Chr13:93.9–94.6
11864	Central nervous system, behavior: Novel open field behavior, vertical activity (rears) from 15 to 30 min for males and females [n beam breaks]	64	39	Chr13:74.4–94.6
11865	Central nervous system, behavior: Novel open field behavior, vertical activity (rears) from min 30–45 for males and females [n beam breaks]	64	34	Chr13:74.2–84.0
11866	Central nervous system, behavior: Novel open field behavior, vertical activity (rears) from 45 to 60 min for males and females [n beam breaks]	64	2	Chr9:73.3–74.0
			4	Chr9:77.2–78.0
			40	Chr13:73.9–84.0
11869	Central nervous system, behavior: Novel open field behavior, vertical activity (rears) in the center from 0 to 60 min for males and females [n beam breaks]	64	39	Chr13:74.0–84.0
12042	Central nervous system, behavior: Novel open field behavior, vertical activity (rears) in the periphery from 30 to 45 min for males and females [n beam breaks]	64	22	Chr13:75.8–82.0
12043	Central nervous system, behavior: Novel open field behavior, vertical activity (rears) in the periphery from 45 to 60 min for males and females [n beam breaks]	64	22	Chr13:75.8–82.0
12047	Central nervous system, behavior: Novel open field behavior, vertical activity (rears) in the periphery from 0 to 60 min for males and females [n beam breaks]	64	23	Chr13:75.8–84.0
12050	Central nervous system, behavior: Saline control response (10 ml/kg), open field behavior, vertical activity (rears) 0–60 min after injection for males and females [n beam breaks/60 min]	64	23	Chr13:75.2–82.0
12063	Central nervous system, behavior: Saline control response (10 mg/kg ip), open field behavior, vertical activity (rears) 0–15 min after injection for males and females [n beam breaks/15 min]	64	23	Chr13:75.8–84.0
12064	Central nervous system, behavior: Saline control response (10 mg/kg ip), open field behavior, vertical activity (rears) 15–30 min after injection for males and females [n beam breaks/15 min]	64	16	Chr13:75.8–79.0
12065	Central nervous system, behavior: Saline control response (10 mg/kg ip), open field behavior, vertical activity (rears) 30–45 min after injection for males and females [n beam breaks/15 min]	64	16	Chr13:75.8–79.0
12066	Central nervous system, behavior: Saline control response (10 mg/kg ip), open field behavior, vertical activity (rears) 45–60 min after injection for males and females [n beam breaks/15 min]	64	14	Chr13:75.8–78.2

*GeneNetwork ID, phenotype and number of lines phenotyped are from GeneNetwork, number of genes refers to the number of genes within the QTL which have human homologs. Position identifies the chromosome and megabase pair (Mbp) location of the gene on mm9*.

Further, we investigated the conservation of the amino acid sequence in the proteins which our candidate genes code for using Protein BLAST. This revealed that the proteins are conserved between mouse and human (RXRG 99% identical; TNR 95% identical; MCTP1 90% identical; CMYA5 68% identical). The strong conservation of RXRG, TNR and MCTP1 supports that they may have an evolutionarily conserved function.

### Gene expression in the brain

The Allen Brain Atlas indicates that *Rxrg* and *Tnr* are expressed throughout the mouse brain (Lein et al., 2007) but that *Cmya5* has high expression only in the cerebellum. For *Mctp1* no area shows strong expression in the mouse brain atlas. Human RNA-*seq* data from BrainSpan (Miller et al., 2014), however, suggests an increase in expression of *MCTP1* in the striatum. *TNR* appears to have a similar level of expression throughout the brain. Interestingly, *RXRG* also shows high expression in the striatum while *CMYA5* shows low expression levels throughout the brain.

### “Guilt-by-association”

Next we used GeneFriends to identify genes which commonly coexpress with our candidates, irrespective of tissue or treatment. GeneFriends uses the gene expression omnibus database and calculates a coexpression value and a *p*-value for this coexpression. Coexpression of a gene with two of our candidates suggests it is involved in their shared function (as a single gene may have several functions, only some of which are shared). We can then use WebGestalt to look for enriched annotations in these coexpressed genes, which may indicate how our candidate genes function to influence BD. *CMYA5* had no commonly coexpressed genes. However, *MCTP1* and *RXRG* have 13 commonly coexpressed genes (Supplementary Table 2), which are significantly enriched for relevant disease annotations (e.g., “Brain diseases” and “Mental disorders”; Supplementary Table 3) and several relevant GO annotations (e.g., “Synaptic transmission”; Supplementary Table 4).

*RXRG* and *TNR* share 797 commonly coexpressed genes (Supplementary Table 5). This shows enrichment for 10 diseases, all of which are highly relevant to neuropsychiatric disorders (e.g., “Substance-related disorders” and “Mental disorders”; Supplementary Table 6). GO and pathway annotations (Supplementary Tables 7–10) show a clear relationship to intercellular signaling (e.g., “Synaptic transmission,” “Neuroactive ligand-receptor interaction” and “Signaling by G protein-coupled receptors”). Interestingly, *MCTP1* and *TNR* share no commonly coexpressed genes. This suggests that although genes related to, for example, mental disorders are enriched in the overlap between *TNR* and *RXRG*, and in the overlap between *MCTP1* and *RXRG*, they are not the same genes, i.e., both sets of genes are independently related to mental disorders.

### Coexpression analysis

The GeneFriends analysis suggests that *MCTP1* and *RXRG* coexpress with mental disorder related genes. BrainSpan shows that both *MCTP1* and *RXRG* are expressed in the striatum, and it has been found that striatal expression *of Rxrg* effects depression-like behaviors in mice (Krzyzosiak et al., 2010). Therefore, we analyzed striatal expression of our four candidate genes in GeneNetwork, using the HQF Striatum Exon RMA data (GN163). This data for mouse striatal exon probes shows clear coexpression between *Mctp1*, *Rxrg*, and *Tnr* (Supplementary Table 11). We could not investigate *Cmya5* exons, as no exon probes were available. However, there are seven intronic probes for *Cmya5* which show a significant *cis*-eQTL and correlate strongly with each other (*r* ≥ 0.5). These do not correlate with *Mctp1*, *Rxrg* or *Tnr* in the striatum.

We further investigated if known mental disorder genes, which commonly coexpress with our candidates in the GeneFriends data, also coexpress specifically in the striatum. Therefore, we built a Pearson correlation matrix including our candidate genes (*Cmya5*, *Mctp1*, *Rxrg*, and *Tnr*), genes commonly coexpressed with *MCTP1* and *RXRG* or *TNR* and *RXRG* within the mental disorders and CNS Diseases annotations (Supplementary Tables 3, 6), and 10 genes containing SNPs which have been strongly associated with BD (*SLC6A4, BDNF, DAOA, DTNBP1, NRG1, DISC1, CACNA1C, ANK3, ODZ4, COMT;* Szczepankiewicz, 2013; Mühleisen et al., 2014). Since there is strong evidence for shared genetics between mental disorders it is likely that we will see an overlap (Doherty and Owen, 2014). As above, we only used probes specific to exons, with the exception of *Cmya5*. An identical table was built for the hippocampus, as the same mRNA microarray has been used and therefore probes were directly comparable. Multiple comparisons were corrected for by dividing the *p*-value of the correlation by the number of probes (*n* = 63) targeting our four candidates (*Cmya5, Mctp1, Rxrg, Tnr*). Probes were said to coexpress if they have an *r* ≥ 0.5 or ≤ −0.5 and an adjusted *p* ≤ 0.05 (*p* = 7.94e-4; *r* = 0.554 or *r* = −0.554 in striatum).

The tables for striatum (Supplementary Table 11) and hippocampus (Supplementary Table 12) show that there are many more coexpressing probes in the striatum than in the hippocampus. Genes with probes which consistently coexpress with several probes for the candidate genes are shown in Supplementary Table 13.

Interestingly, *Cmya5* shows no correlation with any of these genes, either in the hippocampus or striatum, indicating that it may be acting in another brain region or during development. The other three genes show strong coexpression with each other, and with other genes related to mental disorders in the striatum, but not in the hippocampus.

### Correlation analysis

Next, we correlated the expression of our four candidates with the behavioral phenotypes shown in Tables 1, 2. This showed a correlation between several *Mctp1* probes (Supplementary Table 11) and the open field test phenotypes (Table 2). We can sum up each of these groups of related traits into two principal components, i.e., the first principal component for the *Mctp1* probes and the first principal component for the open field traits and look at the correlation between these two. The open field PC1 explains ~75% of the variance between traits and the *Mctp1* PC1 explains ~60% of the variance. These two principal components show a Pearson's correlation of 0.562, *p* = 1.86e-3. This suggests that there is a link between expression level of *Mctp1* in the striatum and vertical activity in the open field test.

We next investigated Pearson correlations between expression of our candidates and the GeneNetwork phenotype database. Each of the four candidates has multiple probes for striatal expression, which were correlated against each other. We then selected those probes that showed a significant correlation (*r* ≥ 0.5) with at least one other probe (Supplementary Table 11). These selected probes were then correlated against the GeneNetwork database of phenotypes. Supplementary Table 14 shows that the expression of *Mctp1*, *Rxrg* and *Tnr* correlates with dopamine related gene expression in the striatum. Further, we found that *Cmya5* expression correlates with anxiety-like and depression-like behavior (Supplementary Table 14). Interestingly, when these phenotypes were correlated against our target phenotypes (i.e., open field test and elevated zero maze traits; Supplementary Table 15), there was no significant correlation with dopamine related gene expression. However, this may be due to a much lower number of overlapping samples (*n* = 27 rather than *n* = 63).

These results suggest that *Mctp1*, *Rxrg*, and *Tnr* may be acting within the same striatal dopamine network, whereas *Cmya5* is acting elsewhere, but still has an influence on anxiety and depression related phenotypes.

## Discussion

This study identified four candidate genes associated with anxiety in mice and BD in humans, *CMYA5*, *MCTP1*, *RXRG*, and *TNR*. The association between *TNR* and BD is a novel finding. In mice, three of our candidates (*Mctp1*, *Rxrg*, and *Tnr*) coexpress in the striatum with other genes related to mental disorders. This suggests that these three genes are part of a pathway which is shared between neuropsychiatric disorders, and which involves the striatum.

Interval mapping in the BXD mouse set produces two regions, within which the QTL for the open field traits (34 measures) and the elevated zero maze traits (7 measures) are located (open field at Chr1:156.2–171.5 Mbp and elevated maze at Chr13:73–97 Mbp). Our results suggest that the QTL defining these two regions are actually made up of two loci rather than one. Firstly the QTL for some traits are found only in a small part of each region, or have two significant loci separated by a non-significant region (Tables 1, 2; Figure 3). Secondly, we find one gene associated with BD at each end of these two regions.

Distal chromosome 1 has been linked to a wide range of phenotypes, many of them neuropsychiatric related (Flint et al., 1995; Gershenfeld et al., 1997; Wehner et al., 1997; Turri et al., 2001a,b; Talbot et al., 2003; Yalcin et al., 2004; Singer et al., 2005; Valdar et al., 2006; Ponder et al., 2007; Mozhui et al., 2008; Eisener-Dorman et al., 2010; Vogel et al., 2013). Genes influencing related phenotypes can often collocate in the genome (e.g., Legare et al., 2000). Often, when a trait is mapped to a single QTL, the locus actually contains several QTL, each with a small effect size (Flint et al., 2005; Valdar et al., 2006). Therefore, our finding of an elevated zero maze QTL on chromosome 1 fits well with the literature.

We find that our genes have previously been associated with schizophrenia, which agrees with an identified overlap in the phenotypes and genetics underlying the two disorders, as well as other psychiatric disorders (Craddock et al., 2006; Owen et al., 2007; Lichtenstein et al., 2009; Purcell et al., 2009; Lee et al., 2013; Cardno and Owen, 2014; Gratten et al., 2014; Pearlson and Ford, 2014; Ruderfer et al., 2014). All four candidates have been associated generally with neuropsychiatric disorders, or psychiatric-like behavior, before. Mouse *Tnr* knock-outs have decreased motivation and increased anxiety (Freitag et al., 2003), a depression-like phenotype. Further *TNR* appears in a GWAS of efficacy of an antipsychotic, iloperidone, for treating schizophrenia (Lavedan et al., 2009). There is some previous association between *RXRG* and BD, as well as other disorders, such as schizophrenia (Le-Niculescu et al., 2008, 2009). *RXRG* has been associated with sensation seeking in humans (Alliey-Rodriguez et al., 2011) and ablation of *Rxrg* in mice leads to depression-like behavior (Krzyzosiak et al., 2010). *CMYA5* has been associated with schizophrenia (Wang et al., 2014; Watanabe et al., 2014), depression (Wang et al., 2014) and BD (Nurnberger et al., 2014).*CMYA5* interacts with dysbindin (Benson et al., 2004), which has been linked to both schizophrenia and BD (Breen et al., 2006; Joo et al., 2007; Pae et al., 2007). *MCTP1* has already been suggested to be associated with BD, but this result was non-significant in their data (Scott et al., 2009).

Finally we show that three of our four genes (*Mctp1*, *Rxrg*, and *Tnr)* coexpress with other genes known to be involved in mental disorders in the striatum, but not in the hippocampus, of adult BXD mice.

### Biological function of candidate genes

Our evidence identifies the striatum as a point of convergence between three of our candidate genes, *MCTP1*, *RXRG*, and *TNR*. The striatum is a relevant brain region in BD, since the diagnostic symptoms of mania include increased risk-taking and deficits in goal regulation (Johnson, 2005; Goodwin and Sachs, 2010), and the striatum is involved in both of these (Balleine and O'Doherty, 2010; Bartra et al., 2013; Mason et al., 2014). Indeed, when making choices about risky decisions, BD patients have increased activity in the nucleus accumbens, part of the ventral striatum (Mason et al., 2014). This may relate back to the mouse phenotypes, as they have to balance the rewards of exploring an exposed area (e.g., mating opportunities) against the risks (e.g., predation), a system that is necessary for all animals and therefore likely to be conserved.

### RXRG

*Rxrg* knockout mice show a reduction in ambulatory activity (Krezel et al., 1998), accompanied by reduction in striatal dopamine receptor expression (Krezel et al., 1998; Krzyzosiak et al., 2010). This alteration is due to direct transcriptional regulation of *Drd2* by retinoid receptors, such as RXRG (Samad et al., 1997; Krezel et al., 1998; Krzyzosiak et al., 2010). In another *Rxrg* knockout, expression of choline acetyltransferase in striatal cholinergic interneurons was reduced and response to antipsychotic dopamine antagonists was altered (Saga et al., 1999).

These studies show that loss of RXRG signaling leads to depression-like behavior in mice, and indicates that decreased dopamine signaling in the striatum plays a critical role in this (Krzyzosiak et al., 2010). Additionally, striatal dopamine receptor expression is clearly involved in inhibition and behavioral control (Lawrence et al., 1998; Cropley et al., 2006; Dalley et al., 2007; Pattij et al., 2007; Hamidovic et al., 2009; Eagle et al., 2011), a trait disrupted in the disorder. This supports that mutations in *RXRG* potentially play a role in BD. Since both *Mctp1* and *Tnr* expression correlated with dopamine traits and with *Rxrg*, it follows that they may be part of this same network.

### MCTP1

*MCTP1* is an understudied gene, however one of the studies has demonstrated that MCTP1 is a calcium binding protein (Shin et al., 2005). It has been suggested that MCTP1's calcium binding properties may be involved in BD (Scott et al., 2009) as other calcium-related genes are associated with BD, such as *CACNA1C* (Ferreira et al., 2008). We find that the calcium signaling pathway is enriched in genes commonly coexpressed with RXRG and TNR (Supplementary Table 8), and disruptions in calcium signaling are part of the pathophysiology of bipolar disorder (Berridge, 2013, 2014). In relation to the above, MCTP1 could be part of an intracellular signaling pathway activated by RXRG or dopamine, or part of a calcium dependent system of regulating dopamine receptor expression, however this hypothesis remains to be tested.

### TNR

*Tnr* knockout mice show decreased motivation to explore and increased anxiety (Freitag et al., 2003). Further, knockout mice spent more time resting and less time eating/drinking (Freitag et al., 2003) and again, activity and appetite are altered in BD (Goodwin and Jamison, 1990). In contrast to this, another *Tnr* knockout experiment showed no increase in anxiety, but increased exploration, although with a different pattern than the wildtype, and an impaired ability to construct a goal-independent representation of space (Montag-Sallaz and Montag, 2003), which may be linked to disruption of goal-orientated behaviors in bipolar patients. These differences may be due to different genetic backgrounds. Freitag et al. backcrossed their mice to C57Bl/6J twice, while Montag-Sallaz and Montag backcrossed to C57Bl/6J 10 times, and therefore may be indicative of epistatic interactions

TNR is a major component of the perineuronal nets (PNNs) of inhibitory interneurons, including those in the striatum (Fuss et al., 1993; Hargus et al., 2008). PNNs are part of the extracellular matrix and ensheath many CNS neurons and their axons and dendritic processes, but not the sites of synaptic contact (Ojima et al., 1995; Alpár et al., 2006; Brückner et al., 2006; Bitanihirwe and Woo, 2014). Specific removal of striatal PNNs in adult mice has direct behavioral consequences (Lee et al., 2012). Further, dysfunction of PNNs has been implicated in schizophrenia (Bitanihirwe and Woo, 2014). In *Tnr* knockout mice the composition and formation of PNNs is significantly altered (Brückner et al., 2000; Haunsø et al., 2000). Therefore, dysregulation of TNR may affect the PNNs of striatal cells and consequently connectivity between cells.

### Role of striatum

Medium spiny neurons are the principal neurons of the striatum. Supplementary Table 14 shows that *Mctp1, Rxrg*, and *Tnr* expression correlates with gene expression signatures for medium spiny neurons. These GABAergic neurons are regulated by glutamatergic and dopaminergic neurons, and presynaptic receptors (including kappa opioid and muscarinic receptors) regulate glutamatergic and dopaminergic transmission (McGinty, 1999). The postsynaptic glutamatergic, dopamine D_1_ and D_2_, and muscarinic receptor signals in the medium spiny neurons trigger a complex intracellular network, resulting in changes in gene and protein expression (McGinty, 1999; Surmeier et al., 2007). In our list of genes which coexpress with *Mctp1*, *Rxrg*, and *Tnr* in the striatum (Supplementary Table 13) we find four GABA receptors, five glutamate receptors and the kappa 1 opioid receptor, further demonstrating a potential link to the above network. Although the full details of the molecular mechanisms remain to be established, especially as different subtypes of striatal neurons express different genes (Surmeier et al., 2007), it is interesting to note that many of these intracellular cascades are Ca^2+^-dependent (McGinty, 1999). The latter may link back to MCTP1 and the role of calcium in bipolar disorder (Berridge, 2013, 2014). Further, medium spiny neurons are a major downstream target of parvalbumin-positive cells, which PNNs particularly colocalize with (Lee et al., 2012).

## Conclusion

The large number of commonly coexpressed genes between *RXRG* and *TNR*, and the enrichment of these genes in mental disorder related annotations, strongly suggests that they are part of the same mental disorder related network. Synaptic transmission related genes are enriched in the commonly coexpressed genes shared between *RXRG* and *MCTP1* and between *RXRG* and *TNR*. Therefore, we hypothesize that disruption of *MCTP1*, *RXRG* or *TNR* alters the complex intercellular signaling within the striatum, leading to changes in intracellular signaling and gene expression. Overall, our study provides evidence for the association of *CMYA5*, *MCTP1*, *RXRG*, and *TNR* with bipolar disorder.

### Conflict of interest statement

The authors declare that the research was conducted in the absence of any commercial or financial relationships that could be construed as a potential conflict of interest.

## Supplementary material

Supplementary Figure 1 **Whole genome QTL maps for all elevated zero maze phenotypes (full details in Table 1)**. Chromosome numbers are shown at the top of each map and megabase pair positions within each chromosome are shown below each map. The higher red line indicates the level of genome-wide significance, i.e., a genome-wide corrected *p* ≤ 0.05, with the blue line showing the significance of the trait at each position. Significance thresholds are shown in the upper left corner, the significant LRS being equivalent to a genome-wide corrected *p* ≤ 0.05. The upper right corner shows the trait ID, as shown in Table 1. The lower red or green line shows the additive coefficient, where a positive additive coefficient (green line) indicates that DBA/2J alleles increase trait values while a negative additive coefficient (red line) indicates that C57BL/6J alleles increase trait values. The scale for the additive coefficient is shown in green on the left of each map.

Supplementary Figure 2 **Whole genome QTL maps for all open field test phenotypes (full details in Table 2)**. Chromosome numbers are shown at the top of each map and megabase pair positions within each chromosome are shown below each map. The higher red line indicates the level of genome-wide significance, i.e., a genome-wide corrected *p* ≤ 0.05, with the blue line showing the significance of the trait at each position. Significance thresholds are shown in the upper left corner, the significant LRS being equivalent to a genome-wide corrected *p* ≤ 0.05. The upper right corner shows the trait ID, as shown in Table 2. The lower red or green line shows the additive coefficient, where a positive additive coefficient (green line) indicates that DBA/2J alleles increase trait values while a negative additive coefficient (red line) indicates that C57BL/6J alleles increase trait values. The scale for the additive coefficient is shown in green on the left of each map.
