# Supplementary figures and images for: A cross-species genetic analysis identifies candidate genes for mouse anxiety and human bipolar disorder
Source: Front Behav Neurosci. 2015 Jul 1;9:171. doi: 10.3389/fnbeh.2015.00171 (PMC4486840; doi:10.3389/fnbeh.2015.00171)

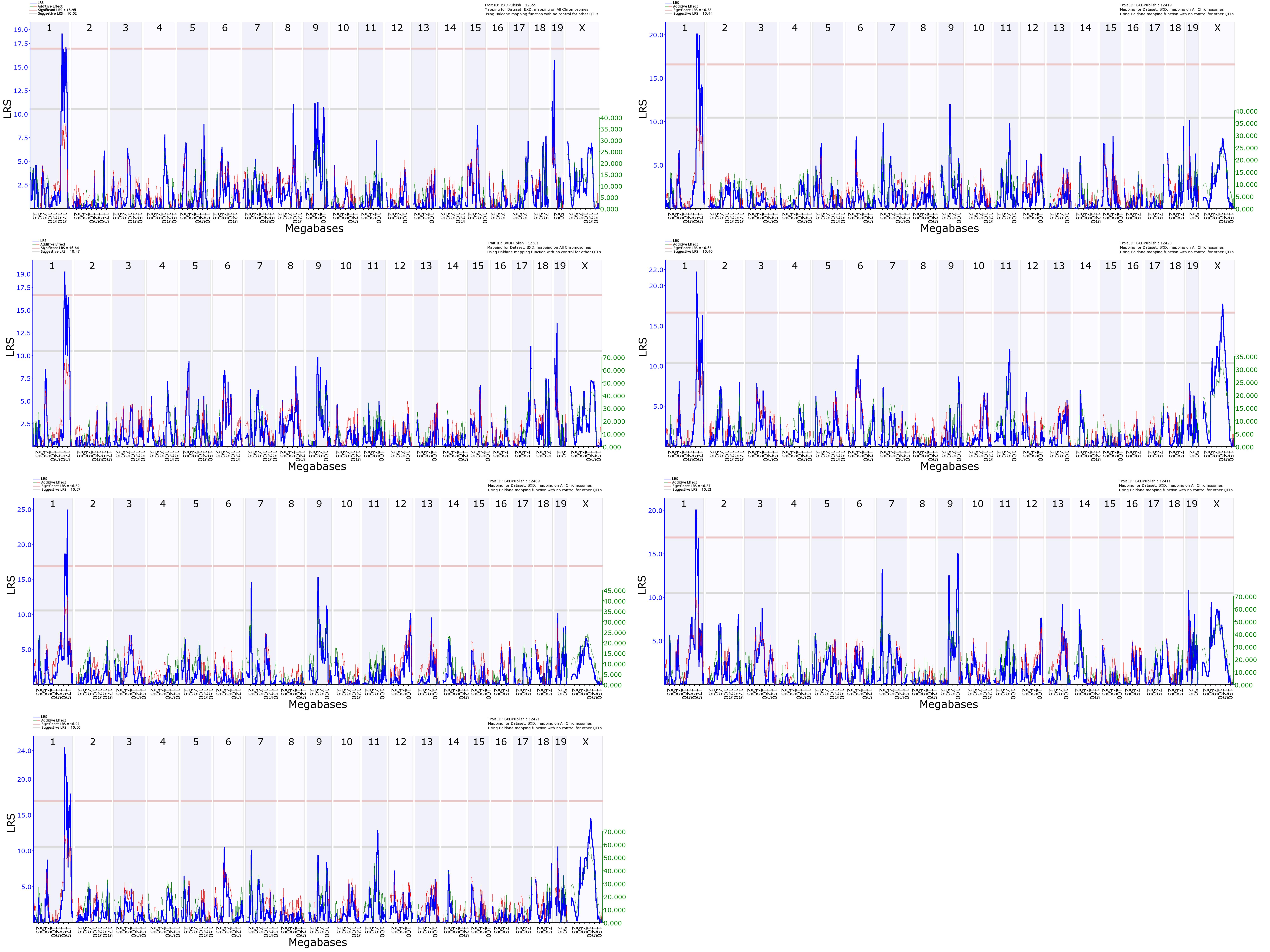

Supplement: Supplementary Figure 1 — Whole genome QTL maps for all elevated zero maze phenotypes (full details in Table 1). Chromosome numbers are shown at the top of each map and megabase pair positions within each chromosome are shown below each map. The higher red line indicates the level of genome-wide significance, i.e., a genome-wide corrected p ≤ 0.05, with the blue line showing the significance of the trait at each position. Significance thresholds are shown in the upper left corner, the significant LRS being equivalent to a genome-wide corrected p ≤ 0.05. The upper right corner shows the trait ID, as shown in Table 1. The lower red or green line shows the additive coefficient, where a positive additive coefficient (green line) indicates that DBA/2J alleles increase trait values while a negative additive coefficient (red line) indicates that C57BL/6J alleles increase trait values. The scale for the additive coefficient is shown in green on the left of each map. [file Image1.JPEG]
